# Polymorphisms of the 11q23.3 Locus Affect the Risk and Mortality of Coronary Artery Disease

**DOI:** 10.3390/jcm11154532

**Published:** 2022-08-03

**Authors:** Tomasz Iwanicki, Joanna Iwanicka, Anna Balcerzyk-Matić, Tomasz Nowak, Katarzyna Mizia-Stec, Paweł Bańka, Artur Filipecki, Jolanta Krauze, Alicja Jarosz, Sylwia Górczyńska-Kosiorz, Anna Ochalska-Tyka, Iwona Żak, Paweł Niemiec

**Affiliations:** 1 Department of Biochemistry and Medical Genetics, School of Health Sciences in Katowice, Medical University of Silesia, Medyków Street 18, 40-752 Katowice, Poland; jiwanicka@sum.edu.pl (J.I.); abalcerzyk@sum.edu.pl (A.B.-M.); tnowak@sum.edu.pl (T.N.); alicja.jarosz@sum.edu.pl (A.J.); iwona.zak@gmail.com (I.Ż.); pniemiec@sum.edu.pl (P.N.); 2 First Department of Cardiology, School of Medicine in Katowice, Medical University of Silesia, 47 Ziołowa St., 40-635 Katowice, Poland; kmizia-stec@sum.edu.pl (K.M.-S.); pawelbanka19@gmail.com (P.B.); afilipecki@sum.edu.pl (A.F.); 3 1st Department of Cardiac Surgery/2nd Department of Cardiology, American Heart of Poland, S. A. Armii Krajowej Street 101, 43-316 Bielsko-Biala, Poland; jolakra@poczta.fm; 4 Department of Internal Medicine, Diabetes and Nephrology, School of Medicine and Division of Dentistry in Zabrze, Medical University of Silesia, 3 Maja Street 13-15, 41-800 Zabrze, Poland; skosiorz@sum.edu.pl; 5 Regional Centre of Blood Donation and Blood Treatment in Raciborz, Sienkiewicza Street 3, 47-400 Raciborz, Poland; atyka@poczta.neostrada.pl

**Keywords:** biological interactions, coronary artery disease, lipid abnormalities, 11q23.3 region, single nucleotide polymorphism, mortality

## Abstract

Background: The present study aimed to determine whether the polymorphisms of the 11q23.3 locus affect the risk and mortality of coronary artery disease in 5-year and 10-year observations. Methods: The study group consisted of 519 subjects: 276 patients with CAD and 243 blood donors as controls. The genotyping of polymorphisms (rs10750097, rs3741298, and rs1729410) was performed using the TaqMan-PCR method. Survival was defined as the period from the angiographic confirmation of CAD to cardiovascular death, and the endpoint was defined as death from cardiovascular causes. Results: The G allele of the rs1729410 polymorphism increased the risk of CAD (OR = 1.55, *p* = 0.04) and showed a synergistic correlation with overweight/obesity (additive synergy index (SI) = 11.01, *p* < 0.001). The carriers of the GG genotype and over-normative LDL levels increased the risk of CAD by over 12-fold higher than expected (multiplicative synergy index (SIM) = 12.34, *p* < 0.001). In the case of the rs10750097 variant, an effect on mortality was shown in both 5-year and 10-year periods. Conclusion: The results revealed that the rs1729410 polymorphism increases the risk of CAD in synergy with traditional risk factors, and the rs10750097 polymorphism of the 11q23.3 locus affects the risk of death in patients with CAD.

## 1. Introduction

The multifactorial nature of coronary artery disease results from the influence of genetic and environmental factors. Several epidemiological studies show the role of lipid disorders in the development of atherosclerotic disease risk [1,2]. The 11q23.3 locus belongs to the regions associated with dyslipidemias. It includes, among others, four genes encoding apolipoproteins (*APOA1*, *APOA2*, *APOA4*, and *APOA5*) involved in cholesterol homeostasis and lipoprotein metabolism, and the regulatory gene *ZPR1*. Previous GWAS studies have identified the selected polymorphic variants of genes clustered in this region as possibly having significant associations with lipid disorders and coronary heart disease [3]. Moreover, the selected polymorphic variants of the 11q23.3 locus are associated with a high risk of cardiovascular events, the most common cause of morbidity and mortality in patients with rheumatoid arthritis [4,5].

**Figure 1:**
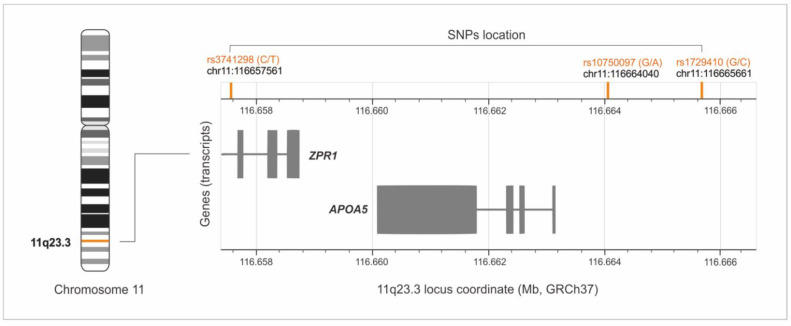
Location of the rs3741298, rs10750097, and rs1729410 SNPs on chromosome 11, at locus 11q23.3 (the figure made with the LDmatrix Tool [11] was adopted).

The haplotype-tagging polymorphisms, namely rs3741298, rs10750097, and rs1729410, are located in the non-coding regions of the 11q23.3 locus (Figure 1), and previous reports indicate their potential regulatory importance [6,7]. It has been shown that the rs10750097 and rs3741298 polymorphisms correlate with changes in serum concentrations of HDL-C and APOA-I [6,7,8]. The rs3741298 polymorphism has also been associated with a reduced risk of coronary artery disease [9]. In some populations, a haplotype containing the rs3741298 variant is considered a genetic risk factor for CAD [3], while the haplotypes comprising the tagging rs1729410 polymorphism have been associated with plasma triglyceride levels in the Chinese population [10].

The aim of the present study was to determine whether the polymorphisms of the 11q23.3 locus (rs10750097, rs3741298, rs1729410) affect the risk and mortality of coronary artery disease. Considering the poorly understood role of selected SNPs, their interactions with the traditional risk factors of CAD as well as possible relationships with the clinical phenotype of the disease were also examined.

## 2. Materials and Methods

This retrospective case–control study was conducted under STROBE guidelines. The case group consisted of patients with coronary artery disease, and the control group included healthy blood donors. Three single nucleotide polymorphisms (SNPs) of the 11q23.3 locus were genotyped. Odds ratio values (OR with their 95% CI) were used as measures of disease risk. The measures of biological interactions between the genetic and traditional risk factors of CAD were Rothman’s additive synergy index (SI), multiplicative synergy index (SIM), relative excess risk due to interaction (RERI), and the proportion attributable to interaction (AP). Mortality of coronary artery disease was analyzed in 5-year and 10-year observations.

### 2.1. Patients and Controls

This case–control study included 491 Caucasian subjects divided into two groups. The first group consisted of 248 patients with angiographically confirmed premature coronary artery disease (CAD group), aged 45.00 (4.00) years. The upper age limit of probands was 55 years. The second group included 243 blood donors (BD group) as control subjects, aged 43.00 (4.00) years, without the presence of symptoms of CAD, myocardial infarction (MI), or stroke, and with no family history of cardiovascular diseases. Survival analysis included a cohort of 276 CAD patients. Patients were recruited from the First Department and Clinic of Cardiology at the Upper Silesian Center of Cardiology in Katowice and the First Department of Cardiac Surgery at the Upper Silesian Center of Cardiology in Katowice. The control group was selected from the blood donors of the Regional Centers of Blood Donation and Blood Treatment in Katowice and Racibórz.

According to the guidelines of the Regional Centers of Blood Donation and Blood Treatment, the control group included only subjects without hypertension and with systolic blood pressure < 140 and diastolic blood pressure < 90 on the day of blood collection. Other detailed inclusion and exclusion criteria were previously described [12]. 

The study groups were recruited between 2001 and 2011. Informed consent was obtained from all individual participants included in the study. This study was approved by the Ethics Committee of the Medical University of Silesia in Katowice (Poland) and informed written consent was obtained from all subjects involved in the study. 

### 2.2. Serum Lipid Measurement

Lipid parameters such as total cholesterol (TC), triglycerides (TG), and HDL cholesterol levels were measured in fresh sera by using enzymatic colorimetric methods (Analco, Warsaw, Poland). LDL cholesterol levels were calculated using the Friedewald formula [13]. 

### 2.3. Genetic Analysis

Genomic DNA was extracted from peripheral leukocytes using a MasterPure Genomic DNA Purification Kit (Epicentre Technologies, Madison, WI, USA). The 11q23.3 locus polymorphisms, namely intergenic variants rs10750097 and rs1729410, and intron variant rs3741298 of the *ZPR1* gene, were genotyped using a TaqMan^®^Pre-designed SNP Genotyping Assay Kit (Applied Biosystems, Foster City, CA, USA). The 20 µL reaction mix consisted of 1 µL template DNA (15 ng/µL), 10 µL TaqMan^®^Genotyping Master Mix (Cat. # 4371355), 1 µL probe (TaqMan^®^ Pre-designed SNP Genotyping Assay), and 8 µL deionized water. The probe was diluted (1:1) in a TE buffer (10 mM Tris-HCl, pH 8.0, 0.1 mM EDTA) before the reaction. PCR was performed according to the manufacturer’s specifications. Genotyping was successful in 90–98% of participants; the accuracy of this analysis was checked by re-genotyping 15% of the samples, and the repeatability of results was 100%. The LDlink Tool (https://ldlink.nci.nih.gov/?tab=ldmatrix, accessed on 27 July 2022) was used to create an interactive matrix of pairwise linkage disequilibrium statistics [11].

### 2.4. Follow-Up and Events

The endpoint in the present study was death from cardiovascular causes, according to the International Statistical Classification of Diseases and Related Health Problems (ICD-10). The complete observation was the occurrence of the endpoint, while the censored observation was the survival or death due to causes other than cardiovascular disease. The data on the date and causes of death of patients (ICD-10) were obtained from the Katowice City Hall and the Central Statistical Office of Poland (Główny Urząd Statystyczny). Survival was defined as the period (in years) from the angiographic confirmation of CAD (which is equivalent to the time of inclusion in the study) until death from cardiovascular causes. 

### 2.5. Statistical Analysis

For data analysis, the Statistica 13.0 (StatSoft, Tulsa, OK, USA) software was used. For quantitative data, the Shapiro–Wilk test was used to check the normality of distribution. A comparison of quantitative variables between CAD and control groups was performed using the Mann–Whitney U test and Kruskal–Wallis test (for variables with non-normal distribution) or Student’s *t*-test (for variables with normal distribution). Beta coefficients (the absolute difference between the median serum lipid concentrations for individual pairs of genotypes) were used to evaluate the differences in lipid concentrations between genotypes. Allele frequencies were deduced from the genotype distributions. The Hardy–Weinberg equilibrium testing, comparisons of genotypes, and allele frequencies between cases and control subjects were calculated using a χ^2^ test in the case of univariate analysis. Statistical significance was accepted at *p* < 0.05. In the case of the analyses of biological interactions, the Bonferroni correction was used, and *p* < 0.025 was considered significant. Odds ratios (ORs with 95% confidence intervals) were computed using univariate and multiple logistic regression analyses after adjustment for age, sex, and the traditional risk factors of CAD. If the number of individuals in the analyzed subgroups was zero, risk ratio values (RR with 95% CI) were used. Association analysis was performed according to three different models: additive; dominant (major allele homozygotes vs. minor allele homozygotes plus the heterozygotes); and recessive model (heterozygotes plus the major allele homozygotes vs. minor allele homozygotes) [14]. The analysis of the survival curves was performed using the Kaplan–Meier estimator. The influence of the assessed parameters on the risk of death was analyzed using the Cox proportional hazard model.

The synergy measures in multiplicative and additive models were used to interpret the extent of interaction, according to the recommendations of Knol et al. [15], and Knol and VanderWeele [16]. Asymmetric confidence intervals (CIs) for additive interaction parameters (SIs) were determined using the model of Zou [17]. Synergy indexes were calculated on the basis of OR values from 4 × 2 tables, using the following formulas:

- For the multiplicative synergy index (SIM):

SIM = OR11/OR01 × OR10,

- For Rothman’s additive synergy index (SI):

SI = OR11 **−** 1/(OR01 **−** 1) + (OR10 − 1),

- For the relative excess risk due to interaction (RERI):

RERI = OR11 − OR10 − OR01 + 1,

- For the proportion attributable to interaction (AP):

AP = RERI/OR11.

## 3. Results

### 3.1. Study Group Characteristics

The clinical and biochemical characteristics of CAD patients and controls are presented in Table 1. CAD patients had increased levels of total cholesterol, LDL cholesterol, and triacylglycerols, as well as higher body mass index (BMI) values and significantly lower HDL cholesterol levels. Furthermore, in the CAD group, there were more nicotine smokers than in the control group (Table 1).

### 3.2. Analysis of the rs1729410, rs10750097, and rs3741298 Polymorphisms in CAD and Control Subjects

The genotypes and allele frequencies of the rs1729410, rs10750097, and rs3741298 polymorphisms are shown in Table 2. All genotype frequencies conformed to the Hardy–Weinberg equilibrium. We decided not to perform a haplotype analysis to determine the possible association of all three polymorphisms with coronary artery disease or its risk factors due to the low R^2^ values indicating independent inheritance in the studied polymorphisms (Table 2).

The frequency of the G allele carriers of rs1729410 was higher in the CAD group than in the control group (*p* = 0.040) compared with the CC homozygotes. The risk of CAD was 1.5-fold higher in G allele carriers. The frequency of other genotypic variants of the analyzed polymorphisms did not differ between the patients and the control group (Table 3).

### 3.3. Analysis of Association between Polymorphisms and CAD Clinical Phenotypes

There were no statistically significant associations between genotypic variants and myocardial infarction, severe atherosclerosis (presence of multivessel coronary disease or critical occlusion > 90%) observed during coronary angiography, left ventricular hypertrophy, and diabetes mellitus (data not shown).

### 3.4. Analysis of Association between Polymorphisms and CAD Lipid Parameters

There was a statistically significant difference in serum HDL levels between the GG and CG carriers of rs1729410 (median ± QD: 1.42 ± 0.34 vs. 1.24 ± 0.35, *p* = 0.021). We did not observe any other association between genotypes and lipid parameters (Supplementary Materials Table S1).

### 3.5. Biological Interactions between Genotypes of Analyzed Polymorphisms and Traditional Risk Factors of CAD

The analysis showed a synergistic interaction between the GG homozygosity of the rs10750097 polymorphism and cigarette smoking, increasing the risk of coronary heart disease (Table 4). The GG cigarette smokers had an estimated risk of CAD more than eight-fold higher than that assumed by multiplicative effects (SIM = 8.91, 95% CI: 1.02–77.48, *p* = 0.047); however, these differences were not statistically significant after the Bonferroni correction. The relative increase in the risk resulting from the interaction between the above factors was positive (RERI = 7.63, 95% CI: −0.77–45.60). The AP value, the combined effect due to the interaction, was 0.72 (95% CI: −0.65–0.81).

Another synergistic interaction was found between the carrier state of the G allele (rs1729410) and overweight/obesity (BMI ≥ 25), significantly increasing the risk of coronary artery disease (Table 5). The carriers of the G allele with overweight/obesity had a CAD risk that was more than 11-fold greater than that resulting from the addition of both effects (SI = 11.01, 95% CI: 2.94–33.31, *p* < 0.001), compared with CC homozygotes. These differences were statistically significant also after the Bonferroni correction. The relative increase in the risk resulting from the interaction between the above factors was positive (RERI = 0.62, 95% CI: −0.65–1.51), and the AP value was 0.37 (95% CI: −0.28–0.87).

Considering the interaction between the over-normative TC levels and the GG homozygosity of the rs1729410 polymorphism (Table 6), the estimated risk of CAD was almost three-fold higher than expected (SIM = 2.91, 95% CI: 1.02–8.34, *p* = 0.047); however, these differences were not statistically significant after the Bonferroni correction. The relative increase in the risk from the interaction between the above factors was positive (RERI = 1.09, 95% CI: −0.50–3.16), and the AP value was 0.37 (95% CI: −0.27–0.66).

Moreover, the results of the interaction analysis of the GG homozygosity (rs1729410) and over-normative LDL levels demonstrated that the risk of CAD was more than 12-fold higher than the risk assumed by multiplication of the effects (SIM = 12.34, 95% CI: 3.51–43.43, *p* < 0.001) (Table 6). These differences were statistically significant after the Bonferroni correction. Additionally, in this case, the relative increase in the risk due to the interaction between factors was positive (RERI = 1.61, 95% CI: 0.70–3.22), and the AP value was 0.80 (95% CI: 0.45–1.15).

### 3.6. Survival and Mortality Analysis of Patients with Coronary Artery Disease

In the case of the 5-year follow-up, 16 patients (5.80%) died, including 14 (5.07%) patients who died of cardiovascular causes (87.50%). The mean survival time of the subjects was 2.44 ± 1.75 years. During the 10-year follow-up, 39 (14.13%) patients died, including 32 patients (11.59%) who died from cardiovascular causes (82.05%). The mean survival time of the patients was 5.22 ± 3.00 years. The cardiovascular causes of death (ICD-10 Classification) are presented in Supplementary Materials Table S2.

At the five-year follow-up, diabetes was the only traditional risk factor that significantly affected cardiovascular mortality (*p* = 0.02). At the 10-year follow-up, none of the analyzed traditional risk factors significantly differentiated patients who died of cardiovascular causes from other patients.

### 3.7. Polymorphisms of the 11q23.3 Locus and Mortality of CAD

**Figure 2:**
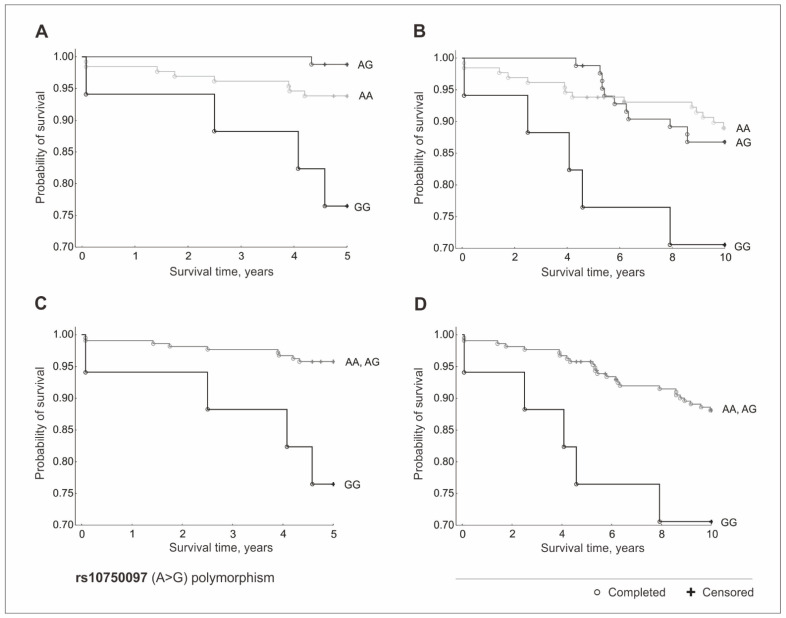
Kaplan–Meier survival curves for CAD patients by rs10750097 (A > G) polymorphism: (**A**) 5-year survival in additive model; (**B**) 10-year survival in additive model; (**C**) 5-year survival in a recessive model; (**D**) 10-year survival in a recessive model.

Out of the three polymorphisms studied, only rs10750097 SNP affected the mortality of coronary artery disease. Over the 5-year period, the AG heterozygotes of the rs10750097 polymorphism were more likely to survive than those of the other genotypes (HR = 0.05, 95% CI = 0.01–0.41, *p* = 0.02). Over the 10-year period, there was a trend toward a greater likelihood of survival with the dose of the A allele, but these differences were not statistically significant. However, significant differences were observed in the recessive model. The GG homozygotes, in comparison to the A allele carriers, were characterized by a 6 times higher risk of death over the 5-year period (HR = 6.01, 95% CI = 1.85–19.52, *p* = 0.003) and an almost 3 times higher risk over the 10-year period (HR = 2.89, 95% CI = 1.11–7.56, *p* = 0.03) (Figure 2).

## 4. Discussion

In the present study, the results indicated the presence of a relationship between the rs1729410 polymorphism and CAD. The G allele carriers had a 1.55 times higher risk of the disease than CC homozygotes. Although the analyzed polymorphisms were not related to the clinical phenotype of CAD, we observed the presence of synergistic interactions between some analyzed genotypic variants and traditional risk factors. The carrier state of the G allele of the rs1729410 polymorphism showed strong synergy with overweight/obesity (BMI ≥ 25). Over-normative BMI values promote inflammatory-mediated diseases, including cardiovascular diseases. The imbalance of pro- and anti-inflammatory systems in obese individuals is reflected in the increased concentrations of CRP protein [18], pro-inflammatory cytokines [19,20], and systemic oxidative stress [21]. Moreover, the GG homozygosity showed synergy with over-normative LDL concentrations, confirming the role of lipid disorders in the pathogenesis of CAD [22]. Our results introduce a certain element of novelty, because so far only a few works on this polymorphic variant have been published, and their results are inconclusive. Previous studies suggest that the intergenic rs1729410 polymorphism belongs to a haplotype block (rs662799, rs17120035, rs9804646, rs1729410, and rs633389) regulating the plasma triacylglycerol levels in patients with metabolic syndrome [10]. However, in the group of Chinese patients, among the analyzed variants of the *APOA1*-*APOA5* gene cluster, no association was found between the discussed polymorphism and familial combined hyperlipidemia [23]. It is speculated that the intergenic variant of rs1729410 has a regulatory role in the genes of the 11q23.3 region; however, further association and functional studies on a larger group are required to clarify its exact significance in the pathomechanism of coronary artery disease.

Our analysis also showed that the rs10750097 polymorphism contributes to the differential survival rates of patients with CAD. The GG homozygotes, in comparison to the A allele carriers, were characterized by a 6 times higher risk of death over the 5-year period and an almost 3 times higher risk over the 10-year period. Additionally, we observed a synergistic association between the GG rs10750097 homozygosity and cigarette smoking, but this result was not statistically significant after the Bonferroni correction, so we can speak of a relative trend rather than a direct association between the analyzed factors. The available literature points to a possible regulatory role of the rs10750097 (A > G) polymorphism of the *APOA5* gene region. The G allele has been reported to be responsible for the formation of the vitamin D receptor nuclear binding site (VDR), which may be responsible for the increase in *APOA5* gene promoter activity [24]. Vitamin D receptors are present in many cells of the human body, including the vascular endothelium, vascular smooth muscle, and immune cells. Through VDR, vitamin D regulates many physiological and pathological processes in cells of the cardiovascular system, such as the growth, migration, and differentiation of vascular cells, the regulation of inflammatory and fibrotic pathways, and the modulation of the immune response. These processes may be involved in the formation of atherosclerotic lesions, from the early stages of vascular endothelial dysfunction to plaque rupture [25]. Additionally, it has been shown that the G allele is a genetic marker modulating the plasma concentration of TG in some populations [26,27] and the factor regulating the body’s response to combination therapy with statins and fibrates in patients with mixed dyslipidemia [6]. The G allele has also been identified as a risk factor for myocardial infarction in the Pakistani population [28]. 

We also observed an association between the GG homozygotes of rs1724910 and increased plasma HDL levels compared with CG heterozygotes (*p* = 0.021). Rs 1729410 was previously analyzed in the context of metabolic syndrome and serum lipid markers, but there were no significant associations between the genotypes of rs1729410, metabolic syndrome, and HDL-C [29]. These inconsistencies may be due to the different ethnicities [6,7,9,10,23,29], different phenotypes (mixed dyslipidemia, familial combined hyperlipidemia) [6,22], different inclusion/exclusion criteria [7,9,10,29], or methodologies (family-based or cohort analyses) [7,9] considered in these studies. 

It should be noted that our study has some limitations. The relatively small groups and their additional division according to 4 × 2 contingency tables reduced the statistical power of the analysis. The small size of our study group may have also influenced the lack of statistical significance for rs10750097 and rs3741298 SNPs and the differences between our results and those of the previous analyses performed with large datasets. Nevertheless, our group was homogenous both ethnically, as well as in terms of sex and age. Large datasets often are not characterized by similar homogeneity. Another limitation is the definition of nicotinism adopted in the study (current state declared by patients during hospitalization), which does not consider detailed information on the dose and duration of exposure to cigarette smoke.

## 5. Conclusions

In conclusion, our results provide support for the hypothesis that the selected polymorphisms of the 11q23.3 locus may affect the risk and mortality of coronary artery disease in Caucasians. In particular, the genotypic variants of the rs1729410 polymorphism increase the risk of CAD in synergy with the common traditional risk factors of CAD such as overweight/obesity and lipid abnormalities. The rs10750097 polymorphism of the *APOA5* gene region affects the risk of death in patients with CAD.

## Figures and Tables

**Table 1:** Clinical and biochemical characteristics of coronary artery disease (CAD) patients and controls.

Characteristics	CAD *n* = 248	Controls *n* = 243	OR * (95% CI)	*p*
Age (years), median (QD)	45.00 (4.00)	43.00 (4.00)	-	0.06
Male gender, *n* (%)	165 (66.53)	174 (71.60)	0.79 (0.54–1.16)	0.22
TC (mmol/L), median (QD)	5.71 (0.97)	4.92 (0.74)	-	<10^−6^
HDL (mmol/L), median (QD)	1.04 (0.14)	1.33 (0.39)	-	<10^−6^
LDL (mmol/L), median (QD)	3.81 (0.84)	2.80 (0.77)	-	<10^−6^
TG (mmol/L), median (QD)	1.73 (0.52)	1.29 (0.40)	-	<10^−6^
BMI, mean (SD)	26.60 (2.76)	25.40 (2.27)	-	0.02
Cigarette smoking, *n* (%)	140 (56.45)	66 (27.16)	3.48 (2.38–5.07)	<10^−7^

CAD, coronary artery disease; CI, confidence interval; OR, odds ratio; QD, quartile deviation; TC, total cholesterol; HDL, high-density lipoprotein; LDL, low-density lipoprotein; TG, triglycerides; BMI, body mass index; SD, standard deviation. * Univariate analysis.

**Table 2:** R^2^ values of the linkage disequilibrium from the CEU population (based on the results obtained with the LDlink tool [11]).

SNPs	rs3741298	rs10750097	rs1729410
rs3741298	1.000	0.314	0.006
rs10750097	0.314	1.000	0.000
rs1729410	0.006	0.000	1.000

**Table 3:** Genotype and allele frequencies of analyzed polymorphisms in patients (CAD) and controls.

Genotype/ Allele	CAD *n* (%)	Controls *n* (%)	Inheritance Model	OR (95% CI)	*p*
*rs1729410*					
GG	57 (25.00)	56 (23.63)	Dominant, vs. GC + CC	1.07 (0.70–1.65)	0.73
GC	121 (53.07)	109 (45.99)	Additive, vs. GG	1.09 (0.69–1.71)	0.71
CC	50 (21.93)	72 (30.38)	Additive, vs. GG	0.68 (0.41–1.14)	0.15
GC + GG	178 (78.07)	165 (69.62)	Recessive, vs. CC	1.55 (1.02–2.36)	0.04 *
C	221 (48.46)	253 (53.38)	-	0.82 (0.64–1.06)	0.13
G	235 (51.54)	221 (46.62)	-	1.21 (0.94–1.57)	0.13
*rs10750097*					
AA	128 (56.64)	143 (63.84)	Dominant, vs. AG + GG	0.74 (0.51–1.08)	0.12
AG	84 (37.17)	68 (30.36)	Additive, vs. AA	1.38 (0.93–2.06)	0.11
GG	14 (6.19)	13 (5.80)	Additive, vs. AA	1.20 (0.54–2.65)	0.65
AG + AA	212 (93.80)	211 (94.20)	Recessive, vs. GG	0.93 (0.43–2.03)	0.86
G	112 (24.78)	94 (20.98)	-	1.24 (0.91–1.70)	0.18
A	340 (75.22)	354 (79.02)	-	0.81 (0.59–1.10)	0.18
*rs3741298*					
TT	120 (54.05)	145 (61.44)	Dominant, vs. TC + CC	0.74 (0.51–1.07)	0.10
TC	91 (40.99)	78 (33.05)	Additive, vs. TT	1.41 (0.96–2.08)	0.08
CC	11 (4.96)	13 (5.51)	Additive, vs. TT	1.02 (0.44–2.36)	0.96
TC + TT	211 (95.04)	223 (94.50)	Recessive, vs. CC	1.12 (0.49–2.55)	0.79
C	113 (25.45)	104 (22.03)	-	1.21 (0.89–1.64)	0.23
T	331 (74.55)	368 (77.97)	-	0.83 (0.61–1.12)	0.23

CAD, coronary artery disease; CI, confidence interval; OR, odds ratio. * statistically significant difference.

**Table 4:** Interaction analysis between GG homozygosity of the rs10750097 polymorphism and cigarette smoking.

GG Genotype, rs10750097	Cigarette Smoking	CAD	Controls	OR	95% CI	
		*n*	*n*		Lower Limit	Upper Limit
0	0	85	151	1	-	-
0	1	123	59	3.70	2.46	5.57
1	0	2	11	0.32	0.07	1.49
1	1	12	2	10.66	2.33	48.75

CAD, coronary artery disease; CI, confidence interval; OR, odds ratio.

**Table 5:** Interaction analysis between carriers of the G allele of the rs1729410 polymorphism and over-normative BMI values.

G Allele, rs1729410	BMI ≥ 25	CAD	Controls	OR	95% CI	
		*n*	*n*		Lower Limit	Upper Limit
0	0	25	37	1	-	-
0	1	17	34	0.74	0.34	1.60
1	0	67	75	1.32	0.72	2.42
1	1	99	87	1.68	0.94	3.02

BMI, body mass index; CAD, coronary artery disease; CI, confidence interval; OR, odds ratio.

**Table 6:** Interaction analysis between GG homozygosity of the rs1729410 polymorphism and risk of over-normative lipid values.

GG Genotype, rs1729410	TC ≥ 5 mmol/L	CAD	Controls	OR	95% CI	
		*n*	*n*		Lower Limit	Upper Limit
0	0	57	98	1	-	-
0	1	100	71	2.19	1.43	3.35
1	0	7	29	0.42	0.17	1.01
1	1	46	27	2.95	1.67	5.23
**GG genotype, rs1729410**	**LDL ≥ 3 mmol/L**	**CAD**	**Controls**	**OR**	**95% CI**	
		***n***	***n***		**Lower limit**	**Upper limit**
0	0	41	42	1	-	-
0	1	114	92	1.27	0.76	2.11
1	0	4	32	0.13	0.04	0.39
1	1	47	24	2.01	1.04	3.86

TC, serum total cholesterol concentration; LDL, serum low-density lipoproteins concentration; CAD, coronary artery disease; CI, confidence interval; OR, odds ratio.

## Data Availability

Not applicable.

## Supplementary Materials

The following supporting information can be downloaded at: https://www.mdpi.com/article/10.3390/jcm11154532/s1. Table S1. Associations between genotypes and serum lipid markers. Table S2. Cardiovascular causes of death in 5-year and 10-year follow-up.
