# Supplementary material for: Polymorphisms of the 11q23.3 Locus Affect the Risk and Mortality of Coronary Artery Disease
Source: J Clin Med. 2022 Aug 3;11(15):4532. doi: 10.3390/jcm11154532 (PMC9369758; doi:10.3390/jcm11154532)
Supplement: Supplementary file 1 [file jcm-11-04532-s001.zip › jcm-1832227-supplementary.pdf]

Supplementary Table S1. Associations between genotypes and serum lipid markers.

Characteristic	Genotypes of rs10750097							*β* coefficient<br>*P* value		
	AA		AG		GG					
	Median	±QD	Median	±QD	Median	±QD	Kruskal-Wallis test	AA vs AG	AA vs GG	AG vs GG
TC (mmol/L), median ± QD	5.00	0.80	4.92	0.77	4.74	0.42	0.697	0.08<br>-	0.26<br>-	0.18<br>-
HDL (mmol/L), median ± QD	1.37	0.39	1.29	0.40	1.29	0.14	0.654	0.08<br>-	0.08<br>-	0.00<br>-
LDL (mmol/L), median ± QD	2.80	0.85	3.00	0.63	2.63	0.76	0.523	0.20<br>-	0.17<br>-	0.37<br>-
TG (mmol/L), median ± QD	1.32	0.43	1.21	0.39	1.26	0.39	0.397	0.11<br>-	0.06<br>-	0.05<br>-
	Genotypes of rs3741298							*β* coefficient<br>*P* value		
	CC		CT		TT					
	Median	±QD	Median	±QD	Median	±QD	Kruskal-Wallis test	CC vs CT	CC vs TT	CT vs TT
TC (mmol/L), median ± QD	4.70	0.57	5.00	0.46	4.92	0.75	0.463	0.30<br>-	0.22<br>-	0.08<br>-
HDL (mmol/L), median ± QD	1.37	0.37	1.28	0.42	1.37	0.39	0.456	0.05<br>-	0.00<br>-	0.09<br>-
LDL (mmol/L), median ± QD	2.78	0.83	2.80	0.81	2.82	0.79	0.973	0.02<br>-	0.04<br>-	0.02<br>-
TG (mmol/L), median ± QD	1.32	0.61	1.40	0.41	1.24	0.35	0.100	0.08<br>-	0.08<br>-	0.16<br>-
	Genotypes of rs1729410							*β* coefficient<br>*P* value		
	CC		CG		GG					
	Median	±QD	Median	±QD	Median	±QD	Kruskal-Wallis test	CC vs CG	CC vs GG	CG vs GG
TC (mmol/L), median ±QD	4.87	0.85	4.97	0.74	4.93	0.74	0.811	0.10<br>-	0.06<br>-	0.04<br>-
HDL (mmol/L), median ±QD	1.32	0.40	1.24	0.35	1.42	0.34	0.046 *	0.08<br>0.130	0.10<br>1.000	0.20<br>0.021 *
LDL (mmol/L), median ±QD	2.72	0.98	2.87	0.72	2.70	0.71	0.345	0.15<br>-	0.02<br>-	0.03<br>-
TG (mmol/L), median ±QD	1.15	0.37	1.30	0.37	1.37	0.50	0.078	0.15<br>-	0.22<br>-	0.07<br>-

Legend: TC, total cholesterol; HDL, high density lipoprotein; LDL, low density lipoprotein; TG, triglycerides; QD, Quartile Deviation; *, statistically significant differences ($P < 0.050$); *β*, absolute differences between medians of lipid markers for specific genotypes.

Supplementary Table S2. Cardiovascular causes of death in 5-year and 10-year follow-up.

ICD-10 code	Cause of death	5-year follow-up		10-year follow-up	
		n	%	n	%
I20.8	Ischaemic heart diseases. Other forms of angina pectoris.	0	0,00	1	3,13
I21.0	Ischaemic heart diseases. Acute transmural myocardial infarction of anterior wall.	0	0,00	1	3,13
I21.9	Ischaemic heart diseases. Acute myocardial infarction, unspecified.	6	42,86	9	28,13
I22.9	Ischaemic heart diseases. Subsequent myocardial infarction of unspecified site.	2	14,29	3	9,38
I24.9	Ischaemic heart diseases. Acute ischaemic heart disease, unspecified.	0	0,00	1	3,13
I25.0	Ischaemic heart diseases. Atherosclerotic cardiovascular disease, so described.	1	7,14	1	3,13
I25.1	Ischaemic heart diseases. Atherosclerotic heart disease.	0	0,00	1	3,13
I25.5	Ischaemic heart diseases. Ischaemic cardiomyopathy.	0	0,00	2	6,25
I25.9	Ischaemic heart diseases. Chronic ischaemic heart disease, unspecified.	2	14,29	6	18,75
I46.9	Other forms of heart disease. Cardiac arrest, unspecified.	0	0,00	1	3,13
I50.1	Other forms of heart disease. Left ventricular failure.	1	7,14	1	3,13
I61.6	Cerebrovascular diseases. Intracerebral haemorrhage, multiple localized.	0	0,00	1	3,13
I63.5	Cerebrovascular diseases. Cerebral infarction due to unspecified occlusion or stenosis of cerebral arteries.	1	7,14	1	3,13
I63.9	Cerebrovascular diseases. Cerebral infarction, unspecified.	1	7,14	1	3,13
I70.2	Diseases of arteries, arterioles and capillaries. Atherosclerosis of arteries of extremities.	0	0,00	1	3,13
I70.9	Diseases of arteries, arterioles and capillaries. Generalized and unspecified atherosclerosis.	0	0,00	1	3,13
Σ		14	100,00	32	100,00

ICD-10 – International Statistical Classification of Diseases and Related Health Problem
